# Adjuvant Biological Therapies in Chronic Leg Ulcers

**DOI:** 10.3390/ijms18122561

**Published:** 2017-11-28

**Authors:** Natalia Burgos-Alonso, Igone Lobato, Igone Hernández, Kepa San Sebastian, Begoña Rodríguez, Gontzal Grandes, Isabel Andia

**Affiliations:** 1 Primary Care Research Unit of Bizkaia, BioCruces Health Research Institute, 48014 Bilbao, Spain; natalia.burgos@ehu.es (N.B.-A.); ksansebastian@gmail.com (K.S.S.); gonzalo.grandesodriozola@osakidetza.eus (G.G.); 2 Preventive Medicine and Public Health Department, Faculty of Medicine and Odontology, Universidad del País Vasco/Euskal Herriko Unibertsitatea UPV/EHU, University of the Basque Country, 48940 Lejona, Spain; 3 Enkarterrri-Ezkerraldea-Cruces Health Region, Basque Health Service (Osakidetza), 48903 Barakaldo, Spain; igone.lobatogarcia@osakidetza.eus (I.L.); igone.hernandezcabezas@osakidetza.eus (I.H.); 4 Bilbao-Basurto Health Region, Basque Health Service (Osakidetza), 48014 Bilbao, Spain; mariabegona.rodriguezrodriguez@osakidetza.eus; 5 Regenerative Medicine Laboratory, BioCruces Health Research Institute, Cruces University Hospital, 48903 Barakaldo, Spain

**Keywords:** biological therapies, chronic leg ulcer, platelet rich plasma, bone marrow concentrates, stromal vascular fraction, mesenchymal stromal cells

## Abstract

Current biological treatments for non-healing wounds aim to address the common deviations in healing mechanisms, mainly inflammation, inadequate angiogenesis and reduced synthesis of extracellular matrix. In this context, regenerative medicine strategies, i.e., platelet rich plasmas and mesenchymal stromal cell products, may form part of adjuvant interventions in an integral patient management. We synthesized the clinical experience on ulcer management using these two categories of biological adjuvants. The results of ten controlled trials that are included in this systematic review favor the use of mesenchymal stromal cell based-adjuvants for impaired wound healing, but the number and quality of studies is moderate-low and are complicated by the diversity of biological products. Regarding platelet-derived products, 18 controlled studies investigated their efficacy in chronic wounds in the lower limb, but the heterogeneity of products and protocols hinders clinically meaningful quantitative synthesis. Most patients were diabetic, emphasizing an unmet medical need in this condition. Overall, there is not sufficient evidence to inform routine care, and further clinical research is necessary to realize the full potential of adjuvant regenerative medicine strategies in the management of chronic leg ulcers.

## 1. Introduction

The overwhelming costs of wound care services is rising worldwide [1,2], with the market of wound care products surpassing $15 billion according to Global Industry Analysts [3]. Healing chronic wounds is becoming a major health challenge. In particular, chronic wounds in the lower limb represent the largest fraction, with venous and diabetic foot ulcers (DFU)s accounting for 70–90% of these ulcers [4]. The socioeconomic and biomedical burdens that they represent are worsened by global demographic events, such as the aging population and the pandemic of obesity [5]. The latter is associated with an increased incidence of diabetes and the threat it involves in foot ulcer development. In fact, up to 25% of diabetic patients will develop a foot ulcer with costs ranging from $7439 to $20,622 per episode [6]. Furthermore, ulcer chronicity increases the severity and the costs of these conditions.

Wound care encompasses all elements of wound management, which include the control of underlying conditions, including neuropathy, ischemia, venous hypertension, pressure, and infection. Impaired wound healing can occur even after controlling modifiable risk factors, and adjuvant biological interventions can form part of complex wound management. Actually, prompt wound healing is imperative to prevent irreversible damage. Moreover, the longer it takes to heal an ulcer, the greater the severity and the financial burden [7].

Concepts in wound healing pathophysiology help to determine the choice of therapy and care planning. The involvement of growth factors was acknowledged decades ago, and as a result, recombinant growth factors (rh-GFs) therapies, such as recombinant human epidermal growth factor (rh-EGF) [8], recombinant human fibroblast growth factor (rh-FGF) [9], and recombinant human platelet-derived growth factor (rh-PDGF) [10] have been explored, resulting in specific growth factors in the therapeutic armamentarium, such as Regranex^®^, (becaplermin, rh-PDGF-BB). But, one by one these growth factors cannot fulfill the multiple needs of non-healing tissues. Grounded on a more biomimetic hypothesis, interventions, such as platelet rich plasma (PRP) therapies, are being tested as they deliver a large pool of molecules that are involved in various healing stages, which can be stalled due to different comorbidities. Healing stages susceptible of PRP modulation include hemostasis, inflammation, cell migration and proliferation, extracellular matrix production, and tissue remodeling [11].

Furthermore, the use of cellular products, in order to address a potential deficiency of competent cells, is under scrutiny. Currently, medical devices and protocols are commercially available to prepare different PRP formulations, and cellular products containing a low number of MSCs, i.e., bone marrow concentrates (BMC), and the stromal vascular fraction (SVF) from adipose tissue [12].

In the context of difficult to heal wounds, regenerative medicine strategies, i.e., PRPs and cell products, may form part of adjuvant interventions in an integral patient management; moreover, taking advantage of using patients’ own resources and in order to avoid drug interactions in these otherwise polimedicated patients, it may be possible to prepare PRP, BMC, or SVF for local application in the wound.

Our review aims to synthesize the clinical experience on ulcer management using two categories of biological adjuvants. Firstly, we have explored the use of point of care MSCs’ related products, and second the local application of platelet derived products. The question that we addressed is as follows: is there any mesenchymal stromal cell or platelet-based regenerative therapy that applied locally, either injected in the wound edges and/or applied topically in the wound bed, can help to heal chronic leg ulcers? The results of controlled trials included in this systematic review favor the use of mesenchymal cell based-adjuvants for impaired wound healing, but the number and quality of studies is moderate-low and complicated by the diversity of biological products. Overall, there is not sufficient evidence to inform routine care. There are 18 controlled studies that are investigating the efficacy of platelet-derived products in lower limb chronic wounds, but the heterogeneity of products and protocols hinders clinically meaningful quantitative synthesis.

## 2. Results

**Figure 1:**
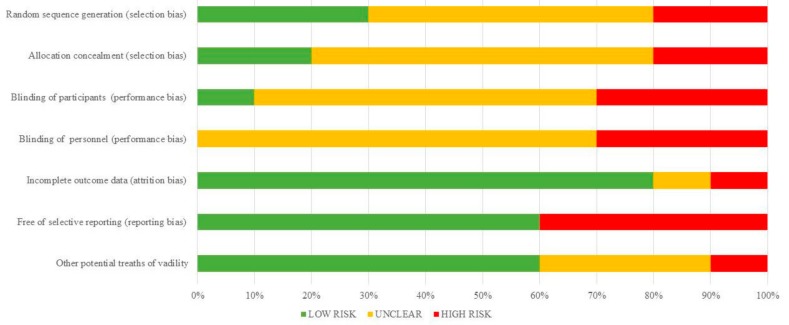
Quantification synthesis of risk of bias (cell therapies).

The search resulted on 813 articles, after removing duplicates. After exclusion of 782 articles for the reasons shown in Figure 1, thirty-one articles remained.

Four articles were excluded after reviewing the full text because they involved the treatment of chronic osteomyelitis, pressure ulcers in the trunk, with various anatomical locations or burn injuries. The main characteristics of cell-based and platelet-based studies are summarized Table 1 and Table 2 [13,14,15,16,17,18,19,20,21,22,23,24,25,26,27,28,29,30,31,32,33,34,35,36,37,38,39,40], respectively.

### 2.1. Cell-Based Studies

Ten controlled studies involving MSC derived therapies were identified, with a total of 261 patients in the experimental arm and 219 patients in the control group [13,14,15,16,17,18,19,20,21,22]. Studies were published between 2005 and 2017. Most studies included DFU [13,14,15,16,17,18,19,20], and one study included ulcers from different etiology (post-trauma, diabetic, arterial, and venous) [21].

There were eight randomized controlled trials (RCTs) [13,15,16,17,18,20,21,22] and two controlled studies [14,19]. The number of patients per experimental group ranged from 10 to 42. Standard wound care or placebo were used as control in most studies. The experimental products tested were: autologous BMC injections [14,16,17,18,20,22], ex vivo expanded bone marrow mesenchymal stromal cells (BM-MSCs) [13,17,18], SVF [15,19,21], and peripheral blood stem cells (PBMNC) after granulocyte colony-stimulating factor (G-CSF) mobilization [14]. Two studies compared two cell products, BMC versus PBMNC [14] and BMC versus BM-MSC enriched in CD90^+^ cells [17]. Whether ex vivo expanded BM-MSCs were superior to BMC was explored in one three armed study [18].

There were no differences between BMC and PBMNCs [14]. When BM-MSCs and BMC were compared [18], the former showed better outcomes, but both cell treatments were better than saline. Two other studies, involving DFU, used SVF combined with fibrinogen/fibrin [15] and with PRP [21].

The patient population was predominantly formed by diabetic patients (in some cases associated with critical limb ischemia [14,18,20]. Main outcome measurements included parameters related to ulcer healing and the rate of amputations. Cell therapies enhanced outcomes in all studies (Table 1).

The risk of bias of cell therapy studies are shown in Table 3 and summarized in Figure 1. The risk of bias arising from the method of generation allocation sequence was considered as low in three trials [15,16,18], five were unclear [13,17,20,21,22], and the two remaining had a high risk of bias [14,19]. The risk of bias arising from the method of allocation concealed was considered low in two trials [13,16]. But in other six studies this was not specified [15,17,18,20,21,22] and two studies did not use adequate allocation concealment [14,19] (Table 3). Concerning performance bias, insufficient description of the blinding procedure was found in six studies [13,16,17,20,21,22]. The remaining three studies had high risk of bias [14,15,19]. The risk of attrition bias was rated as low in seven trials [13,14,15,16,17,20], and was unclear in three studies [18,19,21]. Reporting bias was rated as low in four trials [13,16,17,19].

### 2.2. Platelet-Based Therapies

We identified 18 studies involving the use of platelets or PRP in chronic leg ulcers; they were published between 1986 and 2017 [23,24,25,26,27,28,29,30,31,32,33,34,35,36,37,38,39,40]. A summary of all the studies is shown in Table 2.

The number of participants per group ranged between seven and 59. The number of participants in the experimental and control groups were 408 and 384, respectively. All of them were RCT except three [33,34,40], but most studies were underpowered [24,25,28,30,31,36,37,38]. All of the studies except one [28] were two armed. The biological interventions were highly variable, not only concerning the composition of platelet products, but also the number of applications, and the interval between applications. Topical PRP gel was used in seven studies [23,24,25,26,32,36,37]. In these studies PRP was stimulated with thrombin and/or Ca_2_Cl or calcium gluconate to initiate coagulation and platelet activation in order to obtain platelt rich (PR) gels. The presence of leukocytes in PRPs and the platelet enrichment relative to peripheral blood are hardly described. One study [35] compared PRP that was obtained through double spinning with leukocyte-platelet rich fibrin (L-PRF) that was obtained through single spinning, and found better outcomes in L-PRP treated patients. PDWHF (platelet derived wound healing factors, i.e., platelet secretome) was topically applied in three studies [30,31,40]. Two studies [27,40] used allogeneic platelets. Platelet lysate obtained by freeze/thawing or sonication was used in two studies [38,39]. Knighton et al. used PDWHF mixed with crystalline collagen [30]. The frequency of application varied between twice daily [30], twice weekly [23,36], or weekly [35].

Time to healing or reduction in the ulcer area was the most common outcome measurements. Seven trials involved predominantly diabetic patients [23,26,29,31,32,36,37], while mixed ulcer etiology were involved in the other studies. Outcome results favored experimental treatments in eleven studies [23,24,26,27,28,30,31,32,36,37,40].

**Figure 2:**
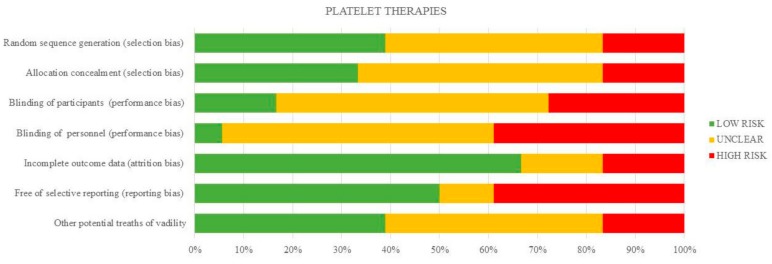
Quantification synthesis of risk of bias (platelet therapies).

The risk of bias for individual studies is shown in Table 4 and the summary is depicted in Figure 2. The risk of bias arising from the method of generation allocation sequence was considered low in eight trials [24,25,26,27,28,31,32,39], and six of these trials had low risk as regards the method of concealed allocation [25,26,30,31,32,39]. Three had a high risk of selection bias [33,36,37]. Overall, most of the studies had a high or unclear risk of performance bias, except for one study [31]. The risk of attrition bias was rated high in three studies [24,26,30], and reporting bias was high in seven studies [28,30,33,35,37,38,40].

## 3. Discussion

We have reviewed controlled studies examining the efficacy of biological adjuvants, mainly cellular products (i.e., BMC and SVF) and platelet-derived products for chronic leg ulcer management. We identified ten controlled studies using mesenchymal-stromal cell based therapies, and 18 studies examining platelet derived products that were applied locally to augment wound healing. Overall, a general positive effect on ulcer size reduction was found in favor of these biological interventions.

Generally, adjuvants are applied when there is a lack of adequate progress on healing. Crucially, DFU precedes 85% of all lower limb amputations [41], thus the importance of developing efficient adjuvant treatments for rapid healing when the ulcer outcome is stalled. As shown in Table 1 and Table 2 summarizing our findings, most patients were diabetic, emphasizing the unmet medical need of this patient population.

Ulcers in the lower extremity may develop from a diversity of conditions, including neuropathy, venous hypertension, mechanical pressure, and ischemia. The latter has been treated with intramuscular injections of either bone marrow derived products, or PBMNCs after mobilization with G-CSF. After systematic review and meta-analysis, data revealed that bone marrow products, but not G-CSF mobilized PBMNCs, improved the surrogate indexes of ischemia [42,43].

There are different strategies to deliver MSCs, from injectable mixtures of cell populations, as is the case with BMC, and SVF, to refined MSC preparations. Most of the studies included in this review used BMC (five studies) or SVF (three studies), which can be prepared at the point of care, using automated closed systems, single use consumables, and clinical grade reagents. These products contain mixed cell phenotypes, in different degree of maturation, including mature cells (adipocytes, fibroblasts, smooth muscle cells, endothelial blood cells and macrophages), progenitors (pre-adipocytes and endothelial, vascular, and hematopoietic progenitors) and stem cells, including MSCs, hematopoietic stem cells, pericytes and supra-adventitial cells [44,45,46]. The rationale for their application is that the non-healing wound is deficient in cells and healing proteins. The main mechanism of action of MSCs consists on paracrine interactions with other cell populations, thereby providing a sustained healing factor delivery to cope with tissue needs [47]. The main mechanisms of action of the mixed cell populations that form part of BMC and SVF have not been described yet.

An advantage of these cellular products is that medical devices and protocols are commercialized to prepare these products (BMC and SVF) at the point of care, facilitating rapid implementation if they were effective. However, our results highlight the low number of treated patients (292), and the great heterogeneity regarding not only cell products, but also outcome measurements, and poor assessment of the power of the study to discriminate the effect sizes of the outcomes; in fact, sample size calculation for at least one clinically important effect is seldom reported [18,22].

Importantly, and relevant to the use of these biological adjuncts for healing, is the fact that no worrying safety concerns were reported in relation to intramuscular injections of this type of therapy [42]. Moreover, the safety of these products in other medical areas, such as intraarticular administration in osteoarthritis, has been ascertained [48,49,50].

Regenerative Medicine treatments are not restricted to cell therapies. There is another category of molecular products obtained from peripheral blood, i.e., PRPs and other platelet therapies. PRP research has increased spectacularly in the last decade. In the 60s, hematologists used PRPs, as a transfusion product for patients with coagulation problems. But, these treatments breathe new life when they were used in the treatment of chronic leg ulcers [51]. These were the first clinical applications of platelets outside of the blood stream, and were followed by the use of PRPs in other medical areas, especially in the field of orthopedics and sports medicine [52,53]. Since then, PRP research has explored the molecular interactions of platelets and plasma secretome with different cell phenotypes [54], thereby constituting a subdiscipline of regenerative medicine. Basic science indicates that PRPs may be promising in the field of wound care, because it enhances cell migration, proliferation, angiogenesis, and tissue anabolism [55]. However, research in novel therapies takes time to develop and optimal indications and protocols are lacking. As we can see in this review, there are different procedures for PRP or PDWHF application (topically or injected), different platelet and leukocyte concentrations, and combination products (i.e., (PRP + collagen powder), (PRP + oxidized cellulose-collagen)). Most studies [23,24,25,26,32,33,36,37] used coagulated PRP, so-called PR gel. These products contain the platelet secretome along with plasma proteins, but activated platelets on their own (without plasma) are also effective [30,31,40].

There are two recent reviews on platelet therapies for wound healing [56,57]. Carter et al. [56] have reviewed and metaanalysed the use of PR-gel on wound healing, acute, and chronic conditions, including prospective and retrospective studies that are published in journals and in congress. The review included 21 studies and nine studies were included in the metaanalysis. The results indicated that PRPs favored healing in chronic ulcers and that the presence of infection was reduced in acute wounds treated with PRP [56]. Martinez-Zapata et al. [57] included 10 RCTs in chronic wounds in their metaanalysis. Three of these RCTs involved DFU and three studies involved venous leg ulcers. Overall analysis did not shed light on PRP effectiveness, but the results indicated that autologous PRP can enhance DFU healing when compared with standard care, although the value of the evidence is low.

Insufficient description of the biological intervention is a major drawback in published studies. At present, the need of minimum reporting standards for biological therapies is recognized [58], and to advance in the field, a consensus regarding minimal requirements for reporting on biological products is obligatory.

PRP helps healing by releasing a physiological pool of proteins involved in different biological processes, including angiogenesis, ECM synthesis, and remodeling. It consists of more than 300 proteins that are released from activated platelets within a fibrin scaffold formed upon plasma coagulation. Alternatively, platelets are used without fibrin. Regarding platelet concentration, PRP containing a near-physiological concentration of platelets (Autologel System, Cytomedix, Gaithersburg, MD, USA) induced a healing response in 96.5% of wounds within 2.2 weeks with 2.8 treatments, in a large observational study using a multicenter registry database (39 centers). The authors concluded that PRP gel could trigger the healing process as positive changes were assessed in 275 of 285 wounds [59,60]. Furthermore, registry data of 26,599 patients, treated between 1988 and 1997 in various Wound Care Centers that are associated with Curative Health Services, showed that platelet releasate was more effective than the standard care, especially in the most severe wounds affecting deeper anatomical structures [61].

Different healing impediments can have varying leverage depending on the specific patient, emphasizing the need to embrace personalized medicine approaches [62]. Healing can be hindered by infection, and osteomyelitis is common in DFU. Sixty-four patients with chronic osteomyelitis randomized to artificial bone implantation with or without autologous PRP and bone marrow implantation showed enhanced bone regeneration in the group with the biological intervention [63].

Importantly, the most suitable dressing to maintain cell viability and optimize the interactions of healing proteins with the ulcer bed has to be defined cautiously [64]. Likewise, needling the fibrotic tissue, while injecting PRP, can eliminate the fibrotic barrier around the ulcer bed, thereby improving healing in selected patients [65].

Based on the current medical literature, it is unclear when these biological adjuvants should be considered, and their place in the decision tree when other treatments are not effective. The need of high quality clinical research is reflected in our review. Although results may be promising, comprehensive reporting that included a clear description of treatment protocols (a description of dressings), providing the composition of the biological product and clinical outcomes, including ulcer size reduction, the time to heal, and the rate of healing are mandatory. Likewise, conducting clinical trials with high quality methodology can help to provide evidence for better clinical care.

## 4. Materials and Methods

We followed the recommendations of the Cochrane Handbook for Systematic Reviews [66] and performed the study according to PRISMA (Preferred Reporting Items for Systematic Reviews and Meta-analysis) statement [67].

### 4.1. Search Strategy

We did a comprehensive systematic search in MEDLINE [68], EMBASE [69], and The Cochrane Library Clinical Trials Database [70] until week 30 July 2017. The search included human clinical studies, written in English. The following algorithm was used to search in MEDLINE via PubMed: (skin ulcer$ OR foot ulcer$ OR diabetic foot OR diabetic feet OR leg ulcer$ OR varicose ulcer$ OR venous ulcer$ OR stasis ulcer$ OR arterial ulcer$ OR neuropathic ulcer$ OR chronic ulcer OR chronic wound) AND (cell OR platelet-derived wound healing factor OR PDWHF OR platelet-rich OR (platelet adj rich) OR “platelet rich plasma” OR “platelet-rich plasma” OR PRP OR “platelet gel$”). We searched through the other data bases with a similar strategy, by using a combination of the terms: “platelet rich plasma”, “skin leg ulcer” and “cell therapy”. We manually checked the references of selected articles to identify additional eligible studies.

### 4.2. Selection Criteria

**Figure 3:**
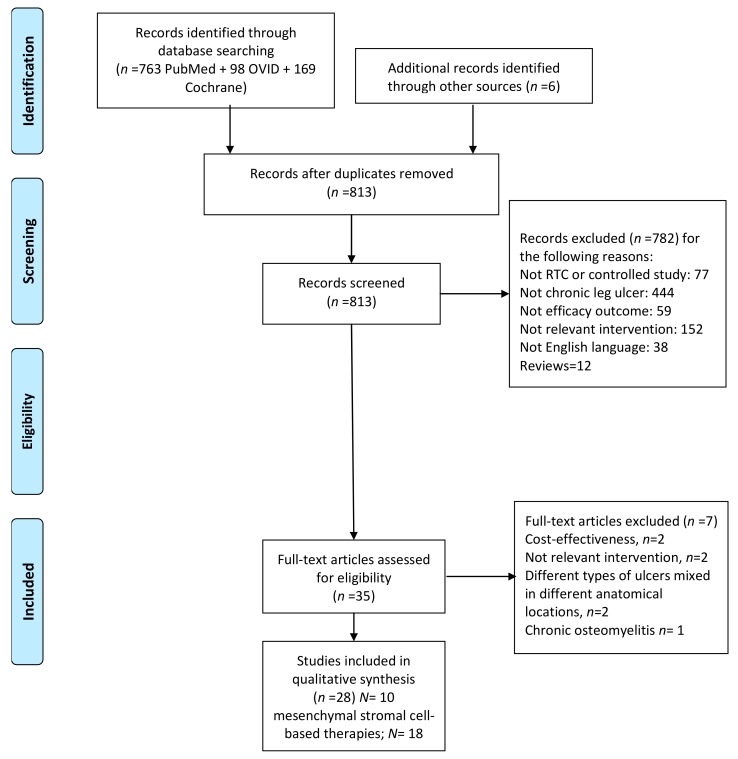
Flow diagram of study selection.

Two reviewers independently assessed each title and abstract of all the articles, and selected a manuscript according to the following criteria: all clinical trials, randomised, and non-randomised comparative cohorts that provided scientific evidence on the efficacy of biological interventions in lower limb ulcers versus other therapies or conventional management were eligible for inclusion. Retrospective observational studies were not included. The experimental treatment had to be any biological adjuvant agent, including mesenchymal stromal cells-based products, i.e., adipose or BMR or MSCs, as biological adjuvants applied locally to the ulcer bed or injected into the edges of the ulcer were included. Studies involving intramuscular injections of mobilized peripheral blood cells or bone marrow derived cells to treat patients with critical limb ischemia (with and without ulcers) were excluded. In addition, we included studies using biological adjuvant agents, such as PRP or PDWHF, (but not recombinant growth factor therapies). Three authors reviewed separately the final list of eligible studies and reach a consensus regarding controversies. (Figure 3).

Results are displayed in two independent Tables: Table 1 summarizes cells’ studies and Table 2 studies involving platelet derived products.

### 4.3. Data Extraction

Full texts were acquired for all of the studies matching inclusion criteria. The following data, related to ulcer healing, were extracted: healing rate, time required for complete wound healing, decrease in the wound area, ulcer size or rate of major amputation relative to the control group. Other extracted data included patient population and pathology, the number of patients in each group, ulcer chronicity, description of the intervention, and control management.

### 4.4. Risk of Bias Assessment

Studies were assessed for quality following the Cochrane Risk of Bias Tool [66].

Two authors independently evaluated the risk of bias of the selected cell intervention studies and two different authors assessed the studies involving platelet therapies, according to Cochrane guidelines.

## Figures and Tables

**Table 1:** Summary of included studies.

Reference (Year)	Study Design Experimental/Control Group	Patient Population	Selection Criteria	Biological Intervention and Control Management	Outcomes/Follow-up	Differences and Statistical Results
Dash (2009) [13]	RCT Exp *N* = 12 CTR *N* = 12	Diabetic foot and Burger disease	Chronicity > 1 month	Exp: BM-MSCs CD90^+^, CD105^+^, CD34^−^, expanded for 5 passages or more CTR: standard wound dressing	Pain-free walking distance and reduction in ulcer size/12 weeks	Cell implant group better than control in pain-free walking and reduction in ulcer area
Dubsky (2013) [14]	Consecutive patients (non-randomized) Three armed Exp1, *N* = 17 patients; Exp2, *N* = 11 patients CTR *N* = 22	Diabetic foot disease	Critical limb ischemia PEDIS 3 TcPO_2_ 30 mmHG or ABI < 0.6	Exp1: BMC Exp2: PBMNC (after G-CSF mobilization) CTR: standard care	Rate of major amputation and TcO_2_/6 months Lost to follow-up: 3	Amputations: 11% in the SCT vs. 50% CTR (*p* = 0.0032) No differences between BMC and PBMNC
Han (2009) [15]	RCT Exp *N* = 26 patients CTR *N* = 26	Diabetic ulcers	Chronicity > 6 weeks	Exp1: SVF+ (fibrinogen/fibrin) C: (fibrinogen/fibrin)	Ulcer size/8 weeks Lost to follow-up: 2	100% complete wound healing in intervention and 62% complete healing in control group
Jain (2011) [16]	RCT Exp, *N* = 25 patients CTR *N* = 23	Chronic lower limb foot in patients with diabetes mellitus	Chronicity > 3 months	Exp: BMC injection CTR: peripheral blood injection	Complete closure Area reduction Wound suitable for surgery	40% ulcer healed in Exp vs. 29% in CTR *p* < 0.05 Area reduction: Exp: 36%/SD0.48 CTR: 27.32% SD0.32 No differences between groups at 3 months Exp *N* = 3 vs. CTR *N* = 1 had skin grafts/3 months Lost to follow-up: 2
Kirana (2012) [17]	RCT Exp 1, *N* = 12 patients Exp 2 *N* = 12 CTR *N* = 6	Diabetic ulcers	Chronicity > 6 weeks	Exp1: BMC Exp2: TRC/BM-MSC expanded enriched in CD90^+^ cells CTR: high n° of drop outs, 4/6 led to exclusion	Complete healing/8 week. Lost to follow-up: 2 Secondary endpoints: time to complete healing, n° major amputation improvement in ABI, TcPO2, BOLD	22 patients received cell treatment. One patient in the TRC group and two in the BMC group did not show wound healing during follow up, 18 patients healed
Lu (2011) [18]	RCT, three armed-study Exp1, *N* = 20 patients; Exp2, *N* = 21 patients CTR: contra-lateral ulcer, *N* = 41	Diabetic patients with CLI and foot ulcer	Bilateral critical limb ischemia (ABI 0.30–0.60)	Exp1: expanded BM-MSC with autologous serum Exp2: BMC CTR: normal saline	Ulcer healing rate, pain at rest and at walking, ABI, TcO_2_, MRA/24 weeks Lost to follow-up: 4	BM-MSC better than BMC in pain at walking (*p* = 0.040), ABI *p* = 0.017, TcO_2_ *p* = 0.001, MRA *p* = 0.018 Cell treated ulcers better than controls in all outcome measures. After 6 weeks the number of healing ulcers in Exp1 was significantly higher than Exp2
Marino (2013) [19]	Cohort study Exp *N* = 10 CTRL *N* = 10	Arteriopathic patients, 18/20 had Diabetes mellitus type 2, five had heart disease and 6 had chronic obstructive pulmonary disease	ABI = 0.3–0.4. all patients underwent revascularization procedure without healing and hyperbaric chamber and oxygen therapy for 6 months	Exp1: SVF, Celution system^®^ (5 mL) cells injected, in all directions, at the edge of the ulcer, depth 1 cm CTR: SVF untreated	Complete closure (primary) Decrease in diameter and depth (secondary)	Follow-up: 4, 10, 20, 60 and 90 days Complete healing in six of 10 patients Four patients did not respond to SVF treatment
Procházka (2010) [20]	RCT Exp *N* = 42 CTR *N* = 54	96 patients with diabetes except 5 in the experimental group; all with CLI and foot ulcer	chronic and critical limb ischemia according to the TASC classification Rutherford 4–6, Fontaine IV	Exp: BMC injection CTR: conventional treatment 40 injections each 1 mL into the ischemic limb	Major limb amputation during 120 days/13 patients died of causes unrelated to therapy	Amputation rate Exp: 21% CTR: 44%
Raposio (2016) [21]	RCT Exp, *N* = 16 patients (21 ulcers) CTR, *N* = 24 patients (31 ulcers)	Chronic skin ulcers (diabetic, post-trauma, arterial, venous)	Ulcer chronicity in the interventional group: 10.19 (SD: 4.37) months and 14.53 (9.75) months in the control group	Exp: ePRP:SVF (mechanical disruption) + PRP (plt: 4–7x) CTR: Standard wound care	Wound closure rate/18 month Lost to follow-up: 0	Exp: 0.2287 cm/day vs. CTR: 0.0890 cm/day (*p* = 0.0257) No matched groups, baseline differences in ulcer area (EXP vs. CTR 29.59 cm^2^ vs. 8.5 cm^2^)
Walter (2011) [22]	RCT Exp *N* = 19 CTR *N* = 21	Aterosclerotic patients	Nonhealing ulcers (Rutherford class 5 or 6)	Exp: autologous bone marrow-derived mononuclear cells (BM-MNC) CTR: Placebo	Complete healing/amputation-free survival/freedom from rest pain Lost to follow-up: 12	Ulcer area decreased significantly in the BM-MNC (*p* < 0.014) but not in CTR group. Patients in CTR group switched to BM-MNC treatment and ulcer area decreased at 3 months. Repeated BM-MNC administration significantly correlated with complete ulcer healing

ABI = ankle brachial index; BM-MNC = bone marrow derived mononuclear cells (isolated through gradient centrifugation); BM-MSC = bone marrow derived mesenchymal stromal cells (purified and expanded ex vivo); BMC = bone marrow concentrate; BOLD = blood oxygen level dependent; CLI = critical limb ischemia; CTR = control group; Exp = Experimental treatment; G-CSF = granulocyte colony stimulating factor; PBMNC = peripheral blood mononuclear cells; PRP = platelet rich plasma; RCT = randomised controlled trial; SVF = stromal vascular fraction; TcO_2_ = transcutaneous oxygen pressure, TRC = tissue regenerative cells.

**Table 2:** Summary of platelet therapy studies.

Author (Year) [Reference]	Study Design Experimental/Control Group	Patient Population	Chronicity of the Ulcer	Biological Intervention and Control Management	Outcomes/Follow-Up	Differences and Statistical Results
Ahmed (2017) [23]	RCT Exp *N* = 28 patients CTR *N* = 28 patients Matched wounds between groups	DFU 56 patients	>6 weeks	Exp: Autologous gelified PRP (4–5x) twice weekly CTR: antiseptic oilment	Ulcer healing, healing rate/8 weeks	Exp: 86% CTR: 68% Healing rate: Exp: 0.7 cm^2^/week CTR: 0.5 cm^2^/week
Anitua (2008) [24]	RCT (pilot) Exp *N* = 8 patients CTR *N* = 7 patients	64% venous, 29% pressure, 7% other Baseline characteristics were not similar between groups	>4 weeks	Exp: Autologous gelified PRP (1.5–2.5x) CTR: Conventional treatment	Mean percentage of surface healed/8 weeks Lost to follow-up: 6	Exp: 5 patients CTR: 4 Exp: 72.94% (SD: 22.25%) CTR: 21.48% (SD: 33.56%) *p* < 0.05
Danielsen (2008) [25]	RCT Exp *N* = 10 patients CTR *N* = 10 patients	Graft surgery in patients with chronic leg ulcers (evaluation of meshed autografts and acute split thickness donor wounds)	Non-reported	Exp: platelet rich fibrin (Vivostat) CTR: saline Platelet rich fibrin sprayed into the donor and recipient wound plus three dressings (two different dressings and one polyurethane closure)	Wound Epithelialization Immunohistomorphometry pain/20 weeks	Epithelial coverage of donor wounds did not differ significantly between platelet-rich fibrin and control on day 5 or day 8
Driver (2006) [26]	RCT Exp *N* = 19 patients CTR *N* = 21 patients	72 patients with type I or II diabetes Efficacy analysis dropouts	>4 weeks	Exp: Platelet gel (autologel^®^) versus CTR: Placebo gel	Proportion of healed ulcers and time to healing 24 weeks	Exp: 13/16 CTR: 8/19 Time to healing shorter in Exp group (*p* = 0.018) 12 week treatment phase Safety evaluation
Jeong (2010) [27]	RCT Exp *N* = 52 patients CTR *N* = 48 patients	DFU	>4 weeks	Exp: Blood Bank Platelet Concentrate versus CTR: treatment with topical fibrinogen and thrombin	Complete wound healing was achieved/12 weeks	Exp: 79% CTR: 46% (*p* < 0.05)
Kakagia (2007) [28]	RCT Exp A *N* = 17 patients Exp B *N* = 17 patients Exp C *N* = 17 patients	51 patients with significant tissue defects of the foot	>3 months	Exp A: oxidized cellulose/collagen Exp B: autologous PRP Exp C: a combination of both	Ulcer dimension within 8 week follow-up	No differences between groups
Karimi (2015) [29]	RCT Exp *N* = 25 patients CTR *N* = 25 patients	DFU	No limit	Exp: PRP CTRL: conventional management	Ulcer’s depth in three weeks	Exp: 4.56 ± 5.76 CTRL: 13.03 ± 14.1 *p* = 0.004
Knighton (1990) [30]	RCT Exp *N* = 16 patients CTR *N* = 16 patients	10 venous diseases, 9 diabetic, 4 occlusive peripheral vascular diseases, and 1 vasculitis	Differences in ulcer chronicity experimental group: 119 days (SD: 114) and control group: 47 days (SD: 63)	Exp: Autologous PDWHF + microcrystalline collagen (Avitene^®^) CTR: placebo (buffer solution + mycrocrystalline collagen) No plasma, platelets resuspended in buffer Topical application	Time to 100% of epithelialization/16 weeks Number of patients analyzed: 13 in PRP group and 11 in control group Lost to follow-up: 2	Exp = 81% vs. CTR = 15% epithelialization *p* < 0.0001
Krupski (1991) [31]	RCT Exp *N* = 10 patients CTR *N* = 8 patients	Number ulcers: 26 Wound aetiology: Mixed 78% diabetic, 72% occlusive peripheral vascular disease, and 28% venous disease	>8 weeks	Exp: PDWHF topical solution) every 12 h CTR: saline solution every 12 h The treatment is applied by the patient	Total epithelialization/12 weeks	Exp: 4/17 CTR: 3/9 Healing rate Exp: −4.3(12.2) cm^2^/week CTR: 1.9 (2.7) cm^2^/week
Li L (2015) [32]	RCT Exp *N* = 59 patients CTR *N* = 58 patients	DFU refractory	>2 weeks	Autologous platelet-rich gel, double spinning and calcium gluconate activation Repeated PR-gel application if reduction of wound area did not reach 80% reduction 2 weeks after treatment	Reduction rate at the end of week 12th/12 weeks Lost to follow-up Exp: 6, CTR: 5	Healing velocity faster in PR-gel group, *p* = 0.020
Moneib (2017) [33]	CT Exp *N* = 20 CTR *N* = 20	Venous leg ulcer Ankle/brachial index > 0.80	>6 months	PR-gel double spinning activation with calcium gluconate + compression CTR: saline management + compression	Reduction in ulcer size expressed as percentage improvement in area	Higher reduction in ulcer size in PRP group compared with control *p* < 0.0001
Obolensky (2017) [34]	CT Exp *N* = 50 patients CTR *N* = 50 patients	Non-healing wounds of different etiology, 82% of the wounds located in lower limb	>6 weeks	Exp: Pure PRP (single spinning) CTR: conventional management	Epithelialization time Hospitalization time Economic effect	Epithelialization: Exp: 42.3 days (SD: 5.7) CTR: 123.8 days (SD: 25.3) Hospitalization Exp 8.4 days (SD: 1.5) CTR: 18.1 (SD: 1.6) €736.81 in savings per patient PRP group
Pravin (2016) [35]	RCT Exp1 *N* = 16 Exp2 *N* = 15	22 venous ulcers, 1 vasculitis, 1 traumatic, 2 diabetic, 4 trophic ulcers	>8 weeks	Exp1: PRP (double spinning) Exp2: L-PRF (single spinning) Weekly administration for 6 weeks	Study period 6 weeks, follow-up 6 weeks	Mean duration of healing: 5.7 weeks in L-PRF and 6.5 weeks in PRP *p* = 0.034
Saad Setta (2011) [36]	RCT Exp *N* = 12 patients CTR *N* = 12 patients	Non healing DFUs	>3 months	Exp: gelified platelet-rich plasma (with bovine thrombin and CaCl_2_) CTR: platelet-poor plasma Treatment applied twice weekly until closure (maximum 20 weeks)	Healing duration in weeks/20 weeks Lost to follow-up: 3	Exp: 11.5 weeks CTR: 17.1 weeks, *p* < 0.005
Saldalamachia (2004) [37]	CT Exp *N* = 7 patients CTR *N* = 7 patients	Diabetic foot 15 patients	>8 weeks	Exp: autologous gelified PRP, topical application CTR: standard care Weekly application for 5 weeks No description of PRP product	Reduction rate = [(final area (mm^2^)—nitial area (mm^2^)/initial area (mm^2^)]/5 weeks Lost to follow-up: 1	Reduction rate 71.9 (22.5) vs. 9.2 (67.8) *p* < 0.039 or reduction of 50% or more was Exp: 71% and CTR: 29%
Sennet (2003) [38]	RCT Exp *N* = 8 patients CTR *N* = 7 patients	Chronic venous leg ulcer	>2 months	Exp: frozen platelet lysate obtained by sonication 10^7^ plt/cm^2^ in saline CTR: saline	Mean percent reduction in ulcer area/12 weeks	Exp: 26.2% CTR: 15.2% (*p* = 0.94).
Stacey (2000) [39]	RCT Exp *N* = 42 patients CTR *N* = 44 patients	Venous ulceration, with no other possible cause for poor healing	>3 months	Exp: autologous platelet lysate (without plasma) 2 × 10^9^ plt/mL CTR: PBS	Ulcer healing/9 months Lost to follow-up: 11	No significant differences between treatment Only ulcer size influenced healing
Steed (1992) [40]	RCT Exp *N* = 7 patients CTR *N* = 6 patients	13 subjects with neurotrophic ulcer	>8 weeks	Exp: PDWHF (obtained from washed allogeneic platelets (without plasma) stimulated with thrombin CTR: placebo	Ulcer healing/Followed for 20 weeks	Exp: 5/7 ulcers healed by week 15th CTR: 1/6 ulcers healed by week 20th

**Abbreviations:** CT = controlled trial; CTR = control group; DFU = diabetic foot ulcer; Exp = Experimental treatment; L-PRF = leukocyte-rich platelet-rich fibrin; PDWHF = platelet derived wound healing formula (or factors); PR (gel) = platelet rich; PRP = platelet rich plasma; PTA = percutaneous transluminal angioplasty; RCT = randomised controlled trial; SD = standard deviation.

**Table 3:** Risk of bias summary for cell therapies studies.

Sources of Bias	Dash (2009) [13]	Dubsky (2013) [14]	Han (2009) [15]	Jain (2011) [16]	Kirana (2012) [17]	Lu (2011) [18]	Marino (2013) [19]	Procházka (2010) [20]	Raposio (2016) [21]	Walter (2011) [22]
Random sequence generation (selection bias)	?	−	+	+	?	+	−	?	?	?
Allocation concealment (selection bias)	+	−	?	+	?	?	−	?	?	?
Blinding of patients (performance bias)	?	−	−	?	?	+	−	?	?	?
Blinding of personnel (performance bias)	?	−	−	?	?	?	−	?	?	?
Incomplete outcome data (attrition bias)	+	+	+	+	+	+	?	+	+	−
Selective reporting (reporting bias)	−	+	+	−	−	+	−	+	+	+
Other bias	+	+	?	+	?	+	?	+	−	+

+: low risk of bias; −: high risk of bias; ?: unclear risk of bias.

**Table 4:** Risk of bias summary for PRP studies.

Sources of bias	Ahmed (2017) [23]	Anitua (2008) [24]	Danielsen (2008) [25]	Driver (2006) [26]	Jeong (2010) [27]	Kakagia (2007) [28]	Karimi (2015) [29]	Knighton (1990) [30]	Krupski (1991) [31]	Li (2015) [32]	Moneib (2017) [33]	Obolenski (2017) [34]	Pravin (2016) [35]	Saad Setta (2011) [36]	Saldalamacchia (2004) [37]	Senet (2003) [38]	Stacey (2000) [39]	Steed (1992) [40]
Random sequence generation (selection bias)	?	+	+	+	+	+	?	?	+	+	−	?	?	−	−	?	+	?
Allocation concealment (selection bias)	?	?	+	+	?	?	?	+	+	+	−	?	?	−	−	?	+	?
Blinding of patients (performance bias)	?	−	?	+	?	?	?	+	+	?	−	?	−	−	−	?	?	?
Blinding of personnel (performance bias)	?	−	?	−	?	?	?	−	+	?	−	?	−	−	−	?	?	?
Incomplete outcome data (attrition bias)	?	−	+	−	?	+	+	−	+	+	+	+	?	+	+	+	+	+
Selective reporting (reporting bias)	+	+	?	+	+	−	+	−	+	+	−	?	−	+	−	−	+	−
Other bias	?	−	+	?	?	?	+	−	+	+	?	?	?	+	?	+	+	−

+: low risk of bias; −: high risk of bias; ?: unclear risk of bias.
